# UMAP-based clustering split for rigorous evaluation of AI models for virtual screening on cancer cell lines*

**DOI:** 10.1186/s13321-025-01039-8

**Published:** 2025-06-10

**Authors:** Qianrong Guo, Saiveth Hernandez-Hernandez, Pedro J. Ballester

**Affiliations:** Department of Bioengineering, Imperial College London, London, SW7 2AZ UK

**Keywords:** Artificial intelligence, Virtual screening, Benchmarking, QSAR, Molecular property prediction

## Abstract

Virtual Screening (VS) of large compound libraries using Artificial Intelligence (AI) models is a highly effective approach for early drug discovery. Data splitting is crucial for benchmarking the performance of such AI models. Traditional random data splits often result in structurally similar molecules in both training and test sets, which conflict with the reality of VS libraries that typically contain structurally diverse compounds. To tackle this challenge, scaffold split, which groups molecules by shared core structure, and Butina clustering, which clusters molecules by chemotypes, have long been used. However, we show that these methods still introduce high similarities between training and test sets, leading to overestimated model performance. Our study examined four representative AI models across 60 NCI-60 datasets, each comprising approximately 33,000–54,000 molecules tested on different cancer cell lines. Each dataset was split in four ways: random, scaffold, Butina clustering and the more realistic Uniform Manifold Approximation and Projection (UMAP) clustering. Using Linear Regression, Random Forest, Transformer-CNN, and GEM, we trained a total of 8400 models and evaluated under four splitting methods. These comprehensive results show that UMAP split provides more challenging and realistic benchmarks for model evaluation, followed by Butina splits, then scaffold splits and closely after random splits. Consequently, we recommend using UMAP splits instead of overly optimistic Butina splits and especially scaffold splits for molecular property prediction, including VS. Lastly, we illustrate how misaligned ROC AUC is with VS goals, despite its common use. The code and datasets for reproducibility are available at https://github.com/Rong830/UMAP_split_for_VS and archived in https://zenodo.org/records/14736486.

**Scientific contribution** This work advances the field by introducing UMAP clustering as a robust splitting method for molecular datasets, improving over traditional methods like Butina clustering and especially scaffold splits. It offers a new evaluation framework to benchmark AI models under more realistic conditions, fostering progress in molecular property prediction. The findings also show how inappropriate the use of ROC AUC for virtual screening (VS) continues to be, despite its popularity, emphasizing the need for context-specific evaluation metrics.

## Introduction

Virtual Screening (VS) has become a key drug discovery technique [1]. VS offers a fast and more cost-efficient method to discover novel molecules that have the potential to bind to a given target (structure-based VS) or, for example, growth inhibition activity on cancer cell lines (phenotype-based VS). Applying computational algorithms and models, VS can significantly reduce the time and financial burdens in the preclinical stages of drug discovery, where it accounts for over 43% of costs in the pharmaceutical industry [2]. Furthermore, VS permits screening gigascale chemical spaces [1] to enable novel drug discovery.

The application of Artificial Intelligence (AI) to improve VS and related problems has tremendous potential [3–14]. Many AI algorithms achieved great performances on popular benchmarks such as those in MoleculeNet [15]. In sharp contrast, only few AI models were able to discover novel molecules with potent whole-cell activity, with few outstanding exceptions [16–18]. This paucity of successful prospective results is partly due to current benchmarks not capturing the challenges posed by real-world screening libraries. This gap highlights the need for improving benchmarks that more accurately represent the complexities of identifying potent drug leads.

The chemical diversity challenge is one of the most important roadblocks to model generalization in drug discovery. Recent studies reported decreases in model prediction accuracy when applied to new drugs compared to new cell lines, illustrating the challenges in extrapolating across diverse chemical spaces [19–23].

To evaluate the model generalizability on dissimilar molecules, many splitting have been proposed to introduce high train-test data dissimilarities, including scaffold split, Butina split and Uniform Manifold Approximation and Projection (UMAP)-based clustering split [24]. The scaffold split was proposed because identifying novel scaffolds is crucial in drug discovery [25]; this method group molecules with the same core structure (scaffold) into the same set [26], thereby offering a more challenging evaluation of model generalizability. The more straightforward method to improve the train-test dissimilarity is the Butina clustering split. This method create dissimilar molecule clusters based on molecular fingerprints through Butina clustering, resulting in non-overlapping clusters of molecules [27]. The UMAP-based clustering split also aligns with these objectives and achieved high clustering qualities in the NCI-60 data by providing a hierarchy clustering on the dimension reduced features to generate dissimilar clusters [24, 28].

While the widely used splitting methods (scaffold and Butina splits) are often regarded as realistic and challenging, e.g. [29], we argue that they do not accurately reflect the real chemical diversity in the compound library to the models. The idea behind these splits is to evaluate the model on molecules with unseen structures, which might benefit model generalization on unseen molecules. However, in vastly diverse libraries such as ZINC [30], the number of unique scaffolds far exceeds those represented in typical training data. As a result, scaffold and Butina splits become less effective because the chemical diversity in the training data is significantly smaller compared to the structures found in libraries such as ZINC20. This limitation prevents those splits in capturing the chemicals diversity in the real world, which reduces its effectiveness in simulating real world situations.

Our analysis demonstrates that the commonly used splitting methods such as random, scaffold and Butina split are not realistic enough to evaluate the model performance. Especially under the scaffold split, molecules with different chemical scaffolds are often similar because such non-identical scaffolds often only differ on a single atom, or one may be a substructure of the other. Surprisingly, this key observation has been overlooked since this split was introduced [15]. Recent work has explored chemically informed splitting protocols that better reflect real-world deployment conditions. For instance, Lo-Hi defines “hit identification” splits based on explicit fingerprint distance thresholds [31], while Tossou et al. propose an out-of-distribution benchmark that aligns test-to-train distances with those between training and deployment libraries [32]. Here we use splits based on UMAP [24] that achieves high-quality cluster separation while maximizing inter-cluster molecular dissimilarity, which introduces more realistic distribution shifts than other splits. In this study, we define a “realistic” split as one that most closely mirrors the chemical dissimilarity found between molecules in modern gigascale compound libraries (e.g., ZINC20 [30] or FDA-approved drugs) and the compounds found in typical training datasets. Chemical similarity is assessed using Tanimoto coefficients on Morgan fingerprints, a standard approach in cheminformatics for quantifying structural resemblance. We consider a split more realistic if the distributional shift it introduces aligns with what would be observed between historical training compounds and novel screening libraries. Furthermore, ROC AUC used as the primary metric in MoleculeNet is a suboptimal metric for VS, as early-recognition performance only makes a small contribution to the ROC AUC, with the rest of contributions coming from less restrictive ranking cutoffs, which thus have absolutely no relevance to VS. The primary metric in VS should instead be hit rate or similar early-recognition metrics, which are aligned to VS goals. To investigate these issues, we employ a benchmark with extensive labeled data across 60 cell lines. Thereby reducing the dataset size as a limiting factor for model performance evaluation in testing our hypothesis against the backdrop of chemical diversity and model generalizability.

This is how this paper extends our preliminary results presented at the ICANN conference. First, we have also evaluated an alternative deep learning model, Transformer-CNN, which applies SMILES-sequence representation learning from natural language processing (NLP). Second, for a better understanding of how easy scaffold splits actually are, we have also added random splits to serve as a performance baseline (there are now 4 splitting methods). Third, we have further refined UMAP splits: instead of simply averaging the results from different held-out folds, we have now merged the predictions from all 7 held-out folds before calculating the metric. Lastly, we refine the employed binarization approach by defining the top 100-ranked molecules as positive predictions instead of the fixed threshold of predicted pGI_50_ > 6 employed at the ICANN study (thus, each binary metric has been recalculated resulting in 1200 new values per metric from the 5 random seeds, 60 datasets and 4 evaluated learning algorithms). This much earlier cutoff (100 molecules with the highest predicted pGI_50_ values) more closely mimics prospective VS tasks, where purchasing many more molecules for in vitro testing is often prohibitive.

## Experimental design

### Datasets

Our study utilized data from the US National Cancer Institute’s NCI-60 cell line screening program [33], released in Oct 2020.^1^ The datasets comprise data of 60 cell lines from nine tumor types: renal cancer, prostate cancer, ovarian cancer, brain cancer, colon cancer, non-small-cell lung cancer, melanoma, breast cancer, and leukemia, to help identify novel anticancer drugs. The growth inhibition conducted by a molecule on a given cell line is summarized by the $GI_{50}$ data for all 60 cell lines. $GI_{50}$ represents the concentration required to inhibit cell growth by 50%, serving as a key indicator of a compound’s potential anticancer activity. The optimal clustering of these molecules [24] revealed seven clusters comprising 33,118 unique molecules across all 60 cell line datasets with 1,764,938 $GI_{50}$ determinations (88.8% completeness). We operate with $pGI_{50}$ (the negative base 10 logarithm of $GI_{50}$ in molar units). Thus, a higher $pGI_{50}$ value corresponds to higher inhibitory potency.

All four splitting methods are applied to the same dataset of 33,118 molecules. As such, the overall chemical diversity is fixed and determined by the composition of the dataset itself. The main difference between these splitting methods lies in how they partition this diversity across different folds. While random and scaffold splits may unintentionally distribute similar molecules within the same fold or fail to reflect global structural variation, UMAP and Butina aim to structure the splits in a way that better represents structural heterogeneity, with UMAP in particular offering a more realistic generalization benchmark.

### Random and scaffold splits

These are the most common data splits nowadays. Before model training, all unique molecules were assigned to a fold index before model training. To match the number of clusters used across different splitting methods, the 33,118 unique molecules were randomly divided into sevenfold, and the molecules were assigned to same fold across all the 60 datasets. This partition was performed by evenly distributing a total of 33,118 unique molecules into seven groups, resulting in an approximate group size of 4731.

A slightly more refined approach is the scaffold split [15, 17], which groups molecules by their core structures, or scaffold, using RDKit’s (https://www.rdkit.org/) Bemis-Murcko method. In this method, molecules sharing the same scaffold are assigned to the same fold, thereby ensuring that the test set contains molecules with entirely different scaffolds from those in the training set. Compared to random splits, scaffold splits are pointed out to be more challenging for models evaluated in chemically diverse datasets [34], as it forces the model to generalize across distinct molecular scaffolds. However, even though this method has limitations; while it ensures distinct scaffolds between sets, it may not fully account for the complexity of real-world molecular diversity, as molecules with different scaffolds can still be chemically very similar.

### Butina and UMAP clustering splits

We employed two clustering-based methods: the Butina split and the UMAP split. These clustering-based methods further facilitate comparisons with the scaffold and Butina splits, as all three methods aim at enhancing the structural diversity between training and test sets, thus offering robust evaluations of model performance in drug discovery applications.

The Butina split clusters molecules based on their molecular fingerprint similarities. Molecules within the same cluster, which share similar chemical features, are assigned to the same fold. While the Butina method introduces more chemical diversity between the training and test sets than random or scaffold splits, it remains limited by the often-low quality of its clusters. Specifically, as shown in a previous study [24], Butina clustering often results in many singleton clusters, and poor performance on standard clustering quality metrics, such as a low (often negative) silhouette coefficient, high Davies–Bouldin scores, and low Calinski–Harabasz scores, indicating weak intra-cluster cohesion and poor inter-cluster separation.

We found the UMAP-clustering split to be optimal for these NCI-60 datasets [24]. UMAP reduces the dimensionality of molecular features, such as Morgan fingerprints, and then uses agglomerative clustering based on their reduced-dimensional features. The optimal setting from the previous study identified seven distinct clusters of the NCI-60 molecules [24]. We previously found that 7 UMAP clusters of these molecules were of higher quality [24] than those produced by Butina clustering [35] and Hierarchical clustering [36]. While dimensionality reduction may raise concerns about information loss, UMAP preserves both local and global structure in the data, making it well-suited for clustering tasks. In fact, the reduced dimensional space often harmonizes inconsistencies in the original high-dimensional Tanimoto space [37], leading to improved clustering quality. In the prior work [24], they compared clustering quality across several methods and found that UMAP-based clustering obtained the highest silhouette scores and best separation between clusters. Thus, dimensionality reduction in UMAP in fact improved clustering quality for our application. Here we define the UMAP split as taking one of these seven clusters as a test set and the rest merged for the training set. Since the number of molecules differs across clusters in the UMAP split, we chose the cluster of 4396 molecules as the test set for the UMAP split for single cell line comparison, which has the closest number of molecules as the test set size in the other splits. After single cell line comparison, we performed sevenfold cross validation under 5 different seeds, and each UMAP cluster was used once as the test set in a sevenfold cross-validation scheme, ensuring balanced evaluation across all clusters despite size imbalance.

Lastly, for a given cell line, only the molecules with an annotated $pGI_{50}$ for that cell line can and are retained in the corresponding data split. For each cell line, this procedure ensures that the training set and test set do not only contain different molecules, but also that their chemical structures are dissimilar.

### Comparative evaluation across the 60 datasets

To evaluate the model efficacy, we performed a sevenfold merged cross-validation, with 5 different seeds for each of the 60 datasets. For cross-validation, we split the data into seven molecular groups. These groups were formed through the traditional splitting methods or by clustering splits. In each cross-validation fold, one group was used as the test set, while the remaining groups served as the training set. In the conference paper [28], we reported performance for each of the seven left-out folds. To evaluate on a larger and more diverse set of molecules, here we merge instead of the predictions from the seven left-out folds and calculate the metric on all the molecules tested on the considered cell line.

### Learning algorithms

We employed four models for comparison: the linear model Linear Regression (LR), the tree-based model Random Forest (RF), the pre-trained graph neural network model (GEM), and the NLP-based Transformer-CNN model.

To predict these $pGI_{50}$ values, two types of input features were employed:

1. Molecular Descriptors. A total of 263 features comprising a Morgan fingerprint (256 bits, radius 2) plus 7 molecular physicochemical descriptors calculated using RDKit [38, 39]. These features were used as input when applying the LR and RF algorithms.
2. Molecular Graphs. Molecular graphs that were constructed from the SMILES strings of the compounds were the input data for GEM [40].
3. SMILES Strings: Transformer-CNN [41] uses SMILES strings as input. The Transformer encoder, pre-trained on SMILES canonicalization tasks, generates dynamic embeddings that capture molecular structure and sequential characteristics of molecules. These embeddings are subsequently processed by the CNN, which applies convolutional filters to extract meaningful latent features.

LR [42] linearly models the relationships between the pre-calculated features and $pGI_{50}$ as the label to predict. The LR model was built using the Python package scikit-learn [43].

RF is a supervised learning algorithm building an ensemble of simple decision trees [44]. When building one decision tree for the RF algorithm, we first pick a subset of the same number of features as the original dataset, with the only difference being that it may include repeated data from the original dataset (bootstrapping). Then, at each training step, the random subset is used as the training set. By repeating the above-described procedure, we will have a variety of decision trees forming an RF model. The RF model makes the predictions by averaging the predicted $pGI_{50}$. This approach that applies bootstrapping and aggregation for predicting is called “bagging”. In this study, we built the RF model for the regression task using the Python package scikit-learn [43].

In addition to these two well-established algorithms, we also used the pre-trained regression model GEM [40], a novel approach that utilizes both molecular topology and geometry information for molecular representation learning. GEM adapts a geometry-based Graph Neural Network (GeoGNN) model pre-trained on 20 million unlabeled 3D molecules from ZINC15 [45], which considers atoms, bonds, and bond angles in a molecule simultaneously. Prospective results on KM-12 [46] show that even a less advanced model than GEM identified a novel scaffold with single-digit µM potency, highlighting GEM’s potential. In our study, we loaded the pre-trained GeoGNN parameters^2^ and then fine-tuned the model for our downstream $pGI_{50}$ prediction task.

The pre-trained GeoGNN model is a graph neural network model used in GEM. It takes in an atom bond graph and a bond angle graph as inputs and outputs the node representation, edge representation, and graph representation. The GeoGNN model consists of several layers of blocks, each of which contains a graph isomorphism network layer, a layer normalization layer, a graph normalization layer, a ReLU activation layer (if specified), and a dropout layer. In each of the blocks, the graph features will be updated through the aggregation process. If $u$ and $v$ are atoms and $\left( {u, v} \right)$ the covalent bonds that connects them, during the model aggregation in the graph, at the $k$-th iteration, the vectors representing the node and edge are $h_{u}$ and $h_{uv}$. $AGGREGATE^{\left( k \right)}$ is the aggregation function in the graph neural networks, which can operate each node neighborhood before using the $COMBINE^{\left( k \right)}$ to combine all the updated node representations. The neighborhood atoms of the atom u are represented as $N\left( u \right)$. The GeoGNN block can be formulated as (adapted from [40]):

$$\alpha_{uv}^{\left( k \right)} = AGGREGATE_{bond - angle}^{\left( k \right)} \left( {\left\{ {\left( {h_{uv}^{{\left( {k - 1} \right)}} ,h_{uw}^{{\left( {k - 1} \right)}} ,x_{wuv} } \right),w \in N\left( u \right)} \right\}} \right)$$

1 $$\cup \left\{ {\left( {h_{uv}^{{\left( {k - 1} \right)}} ,h_{uw}^{{\left( {k - 1} \right)}} ,x_{wuv} } \right),w \in N\left( u \right)} \right\}$$

2 $$h_{uv}^{\left( k \right)} = COMBINE_{bond - angle}^{\left( k \right)} \left( {h_{uv}^{{\left( {k - 1} \right)}} ,\alpha_{uv}^{\left( k \right)} } \right)$$

The GeoGNN model uses mean pooling as the readout layer to obtain the graph representation. The graph pooling layer takes in the atom bond graph, the node representation, and the edge representation as inputs and outputs the final graph representation. After the GeoGNN, it is followed by a Multilayer Perceptron (MLP), which takes the graph representation as input and predicted $pGI_{50}$.

A hybrid model is Transfomer-CNN [41] combining a Transformer-based encoder and a Convolutional Neural Network (CNN). The Transformer encoder, pre-trained on SMILES canonicalization tasks, generates contextualized SMILES embeddings that capture molecular structure and sequential properties. These embeddings serve as inputs to the CNN, which applies multiple convolutional filters with varying kernel sizes to extract high-level features. This layered design enables Transformer-CNN to process sequence data effectively and supports transfer learning [47–50], advancing over earlier QSAR methods [51, 52]. The Transformer-CNN implementation follows the architecture described by [41].

### Test set performance metrics

We assessed model performance using a dual regression-classification approach. Molecules with measured $pGI_{50}$ values larger than 6 ($GI_{50} = 1$ µM) were considered as positives (actives) and below or equal 6 as negatives (inactives) in the NCI-60 dataset. For a given test set, we then identified 100 molecules with the highest predicted $pGI_{50}$ as positives, with all remaining molecules predicted negatives. This ranking-based classification method simulates the process in VS, where only the most promising compounds from large chemical libraries can be selected for further experimental validation in the lab. This mimics the real-world scenario where one can test in vitro 100 molecules in the considered cell line (this is more realistic than selecting the cutoff with p > 6 as done in the conference paper [28], since this often results in too many or too few molecules to test in vitro). By adopting this approach, we categorize each prediction as either True Positive (TP), True Negative (TN), False Positive (FP), or False Negative (FN). Evaluation metrics such as False Positive Rate (FPR) and True Positive Rate (TPR or Sensitivity) were calculated from them. Metrics for VS have been explained elsewhere [53], but for convenience are summarized below.

Hit rate, our primary metric, measures the proportion of TP in compounds identified as positive, as in Eq. 3, which perfectly aligns with the objective of VS: screen vast compound libraries to identify potential leads. In VS, early identification of active compounds (hit rate) is critical, as only a small subset of compounds is chosen for further experimental validation. Unlike Receiver Operating Characteristic (ROC) curve, which treats all classification errors equally, hit rate prioritizes true positives in the top-ranked predictions, aligning better with the practical goals of VS. With a high hit rate, we are more confident in having more efficient results to find promising leads from a pool of candidates.

3 $$hit\,rate\left( \% \right) = \frac{TP}{{TP + FP}} \times 100\%$$

Matthews Correlation Coefficient (MCC) is a metric summarizing both types of errors:

4 $$MCC = \frac{TP \times TN - FP \times FN}{{\sqrt {\left( {TP + FP} \right)\left( {TP + FN} \right)\left( {TN + FP} \right)\left( {TN + FN} \right)} }}$$

The ROC curve illustrates the performance of a binary classifier as its discrimination threshold varies. It is the plot of TPR against FPR. TPR indicates the proportion of TPs being correctly identified as such, while FPR measures the proportion of negatives being incorrectly classified as positives. Thus, the Area under the ROC Curve (AUC) evaluates the model’s ability to distinguish between the classes, which in this case are active and inactive compounds. A higher ROC AUC value indicates better model performance, with a value of 1.0 representing a perfect classifier, and a value of 0.5 indicates no discriminative power (equivalent to random guessing).

Root Mean Square Error (RMSE) is the square root of the mean of the squared differences between the actual $pGI_{50}$ $\left( {y_{i} } \right)$ and the model predicted $pGI_{50}$ $\left( {\hat{y}_{i} } \right)$:

5 $$RMSE = \sqrt {\frac{{\mathop \sum \nolimits_{i = 1}^{n} \left( {y_{i} - \hat{y}_{i} } \right)^{2} }}{n}}$$

### Model hyperparameters

We trained the LR and RF models using the scikit-learn Python package [43]. The models were trained using the default settings, with the hyperparameters provided in Table 1. For GEM, we used the pre-trained GeoGNN model parameters provided by its authors. We fine-tuned the GEM for 100 epochs on our dataset, using the default settings, including a batch size of 32, a dropout rate of 0.1, and a learning rate of 0.001. The Transformer-CNN model followed a similar approach. To ensure comparability with other models while optimizing runtime, we set the training epoch to 5. The Transformer-CNN fine-tuning used a batch size of 32. To enhance the robustness of the Transformer-CNN model, we applied SMILES augmentation during training. Specifically, we used RDKit to generate 10 non-canonical SMILES strings per molecule by randomizing the atom order. For each molecule, hydrogen atoms were first removed and salts were stripped. Next, up to 10 randomized SMILES were created using Chem.MolToSmiles, with the root atom index randomly selected for each iteration. This process results in up to 11 SMILES strings per molecule, depending on whether the molecule passed the preprocessing and had at least one valid atom after standardization. Some molecules generated fewer than 11 SMILES due to invalid structures. During validation and testing, we used only the canonical SMILES for each molecule to ensure consistent and unbiased evaluation.

**Table 1:** Hyperparameters for LR, RF, GEM, and Transformer CNN. The head learning rate is the learning rate for the MLP after the pre-trained GeoGNN, and the encoder learning rate is the learning rate for the pre-trained GeoGNN

Algorithm	Hyperparameter	Default value
LR	fit_intercept	True
RF	n_estimator	100
	Criterion	Squared error
	Max_depth	None
	Min samples split	2
	Min samples leaf	1
	Min weight fraction leaf	0.0
	Max features	1.0
	Max leaf nodes	None
	Min impurity decrease	0.0
	Bootstrap	True
	Max samples	None
GEM	Batch Size	32
	Dropout Rate	0.1
	Encoder Learning Rate	0.001
	Head Learning Rate	0.001
	Epoch	100
Transformer-CNN	Batch Size	32
	Epoch	5
	Canonize	True

For computational efficiency, we benchmarked runtime on the AMD EPYC 7742 64-Core Processor. Each learning algorithm, that is LR, RF, GEM, and Transformer-CNN, was run on this CPU resulting in 8400 models being trained and tested per algorithm. GEM had an average runtime of 347 min per run, while Transformer-CNN completed each run in an average of 306 min. We adjusted the number of Transformer-CNN training epochs to align its runtime more closely with GEM for fair comparison.

## Results

### Limitations of random and scaffold splits in realistic VS

It is widely known that random splits tend to overestimate model performance due to their unrealistically high train-test similarities. Here molecules are assigned to training and test sets randomly, which often results in both sets containing molecules with very similar properties or even slight variations of structures. This leads to overestimated performance, as models effectively “memorize” the dataset rather than generalizing to truly novel compounds.

**Fig. 1:**
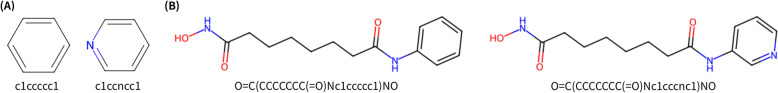
Bemis-Murcko scaffolds can be almost identical, and thus the scaffold split often introduces strong training-test similarities. **a** Benzene and pyridine are the two most frequent scaffolds in the molecules tested on the NCI-60 IGROV1 cell line (each scaffold has its own SMILES string underneath). The scaffold split ensures that the test set only contains molecules with unseen scaffolds, i.e., no molecules in the training set have the same scaffold in the test set. **b** However, there will be molecules with similar scaffolds, which would not all be placed in the same set despite being globally similar, e.g., benzene-containing molecules could be assigned to the test set when pyridine-containing molecules are in the training set. Vorinostat (left) and pyroxamide (right) are similar pairs of molecules with different scaffolds. As a result, the performance of a model trained and evaluated on the scaffold split will be overestimated

We recently showed that the scaffold split, which is widely believed to be a realistic split [15], also overestimates VS performance [28]. This split ensures that the test set only contains molecules with unseen scaffolds. That is, there are no training molecules with any of the scaffolds observed in the test molecules. However, molecules with different scaffold structures can be almost identical. For instance, the 48,416 molecules tested on the IGROV1 cell line by the NCI-60. The two most frequent scaffolds in these molecules are benzene and pyridine (Fig. 1a), which have nearly identical Morgan fingerprints but classified into different scaffolds. Both molecules are almost identical in that they differ in a single atom. Surely, there are cases where a single atom can result in strong bioactivity differences (the well-known activity cliffs). Despite this fact, the similarity principle holds: on average, molecules with similar chemical structure possess substantially more similar properties compared to randomly chosen molecules [54]. The scaffold split can place all benzene-containing molecules in one set and all pyridine-containing molecules in the other set. To illustrate this, consider vorinostat and pyroxamide, two molecules with different Murcko scaffolds but highly similar structures. Despite being assigned to different scaffolds, these molecules exhibit a Pearson correlation of 0.938 in their values across 3 shared targets in release 35 of the ChEMBL Database [55] (Fig. 1b and Table 2). For example, both exhibit strong inhibition of HDAC6 (CHEMBL1865) with $pIC_{50}$ values of 7.429 and 7.700, respectively. This example showed how the scaffold split can assign structurally and functionally similar molecules to different partitions, introducing high train-test similarities and lead to overestimated model performance.

**Table 2:** $pIC_{50}$ values of vorinostat and pyroxamide across their 3 common targets

Target ChEMBL ID	Description	Vorinostat $\left( {pIC_{50} } \right)$	Pyroxamide $\left( {pIC_{50} } \right)$
CHEMBL1865	HDAC6 (Histone deacetylase 6)	7.429	7.700
CHEMBL2093865	HDAC1 (Histone deacetylase)	6.740	5.849
CHEMBL394	HCT-116	5.801	5.187

Despite having different scaffolds, the two compounds exhibit similar activity profiles

The resulting benchmarks will hence not be realistic, as the real test sets, compound libraries for prospective VS, contain not only new, but novel scaffolds [30]. Evaluating models using the scaffold split overestimates their prospective performance, as those real test sets are more complex and challenging. Thus, current benchmarks can miss the most promising models for prospective applications. There is hence an urgent need to investigate the impact of this shortcoming and provide data splits that are closer to the real level of difficulty posed by prospective VS.

### Splitting evaluation and real-world relevance

**Fig. 2:**
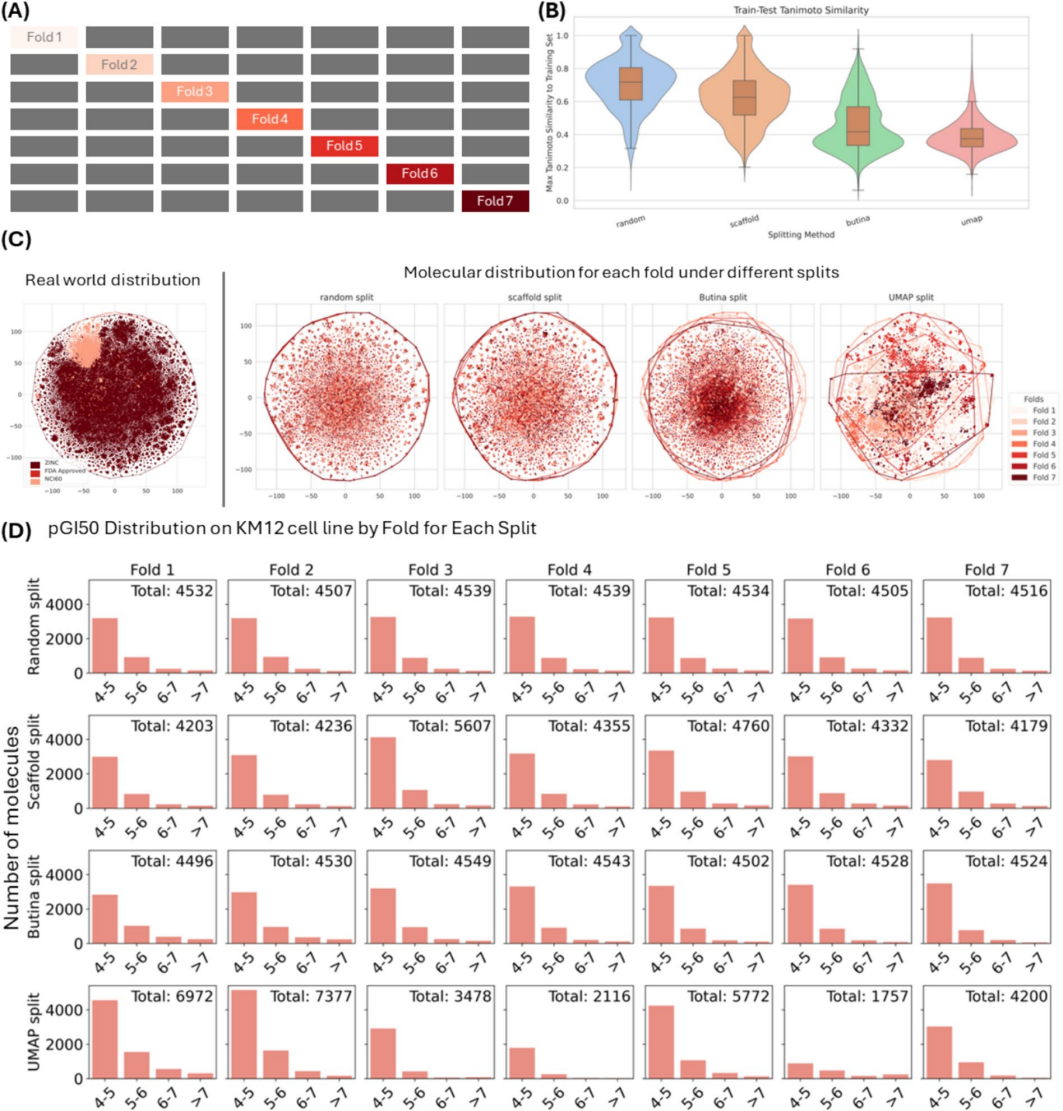
**a** sevenfold cross validation implementation for the NCI-60 dataset. **b** Violin plot of the distribution of maximum Tanimoto similarity between test molecules and their nearest training set molecule across the 60 datasets for each splitting method. For each test molecule, we computed its 2048 bits and 2 radius Morgan fingerprint and measured the Tanimoto similarity to all molecules in the training set. We then recorded the maximum similarity score for each test molecule, representing the most similar compound in the training set. Lower similarity values indicate greater distributional shift. **c** t-SNE molecular fingerprints projections for chemical space coverage and boundaries: the leftmost panel shows molecules from two real-world sources and our training data (30 million ZINC compounds and 33,118 NCI-60 molecules). The remaining four panels are each splitting strategy partitions in this space for a representative dataset, with points colored by assigned fold. Polygons outline the spatial extent of each fold using convex hulls. **d** The $pGI_{50}$ value distribution across folds for the KM12 under different splitting methods. Each row represents a splitting method: Random, Scaffold, Butina, and UMAP, while each column corresponds to one of the seven folds. Bars indicate the number of molecules in each $pGI_{50}$ range: 4–5, 5–6, 6–7, and > 7

To quantitatively assess the dissimilarity introduced by each split, we computed the average maximum Tanimoto similarity between each test molecule and its nearest neighbor in the training set. We observed the following means across all 60 datasets: random split (0.717), scaffold split (0.625), Butina split (0.417), and UMAP split (0.375) as shown in Fig. 2b. These results confirm that the UMAP split introduces the largest distributional shift between training and test molecules, validating our characterization of it as the most chemically dissimilar and hence realistic split.

To assess how well each split reflects real screening libraries, we compared the molecular diversity of their test sets with compounds from two real-world sources: the ZINC database [30] (30 million randomly selected molecules) and the FDA-approved drug list [56] (2766 compounds). These libraries are common in practical VS tasks. As shown in Fig. 2c, test folds produced by the UMAP split tend to occupy more distinct regions in the chemical space, resulting in test sets that contain more structurally dissimilar molecules. In the real-world distribution plot, the ZINC molecules span a much broader space than those in the NCI-60 dataset, indicating that most real-world compounds differ from those used during model training. This supports the idea that UMAP splitting better simulates realistic screening conditions, where the model faces unseen and diverse chemical structures.

The splits were performed on the 33,118 unique molecules from NCI-60 dataset. For transparency, we report the fold and cluster sizes across all four data splitting methods in the sevenfold cross validation in Table 3. These distributions reflect the varying nature of how each method segments chemical space. In particular, the UMAP and scaffold splits generate variable fold sizes due to the underlying structure of the molecular clusters. We further examined the distribution of activity values $\left( {pGI_{50} } \right)$ within each fold using bins of 4–5, 5–6, 6–7, and > 7 (Fig. 2d). Notably, although UMAP split produces variable cluster sizes, they sometimes result in smaller test sizes than other three splitting methods in fold 3, 4, and 6, which correspondingly lead to larger training sets for these folds. Despite this data advantage, the average hit rate on these UMAP test folds for Transformer-CNN remains lower (33.27%) than that achieved with random (67.67%), scaffold (64.53%), or Butina (43.60%) splits. This suggests that the reduced performance is not due to limited training data but rather reflects a more challenging generalization task caused by stronger distributional shift (Fig. 2b).

**Table 3:** Test fold sizes for the sevenfold splits across four splitting methods

Split	Fold 1	Fold 2	Fold 3	Fold 4	Fold 5	Fold 6	Fold 7
Random	4732	4731	4731	4731	4731	4731	4731
Scaffold	4366	4405	5865	4586	4993	4532	4371
Butina	4731	4731	4731	4731	4731	4731	4732
UMAP	7287	7620	3762	2250	6002	1801	4396

### GEM and RF perform similarly with traditional random and scaffold splits

We first started by comparing GEM to a nonlinear baseline (RF), a linear baseline (LR), and the language-based model Transformer-CNN on the MCF7 cell line using random, scaffold, and Butina split. We adopted a dual regression-classification evaluation approach, classifying molecules from NCI-60 datasets as positive or negative based on a measured $pGI_{50}$ cutoff at $6$, with the top 100 model predictions considered as predicted positives (note that this type of cutoff results in a predicted $pGI_{50}$ cutoff that varies across models, splits and cell lines).

**Fig. 3:**
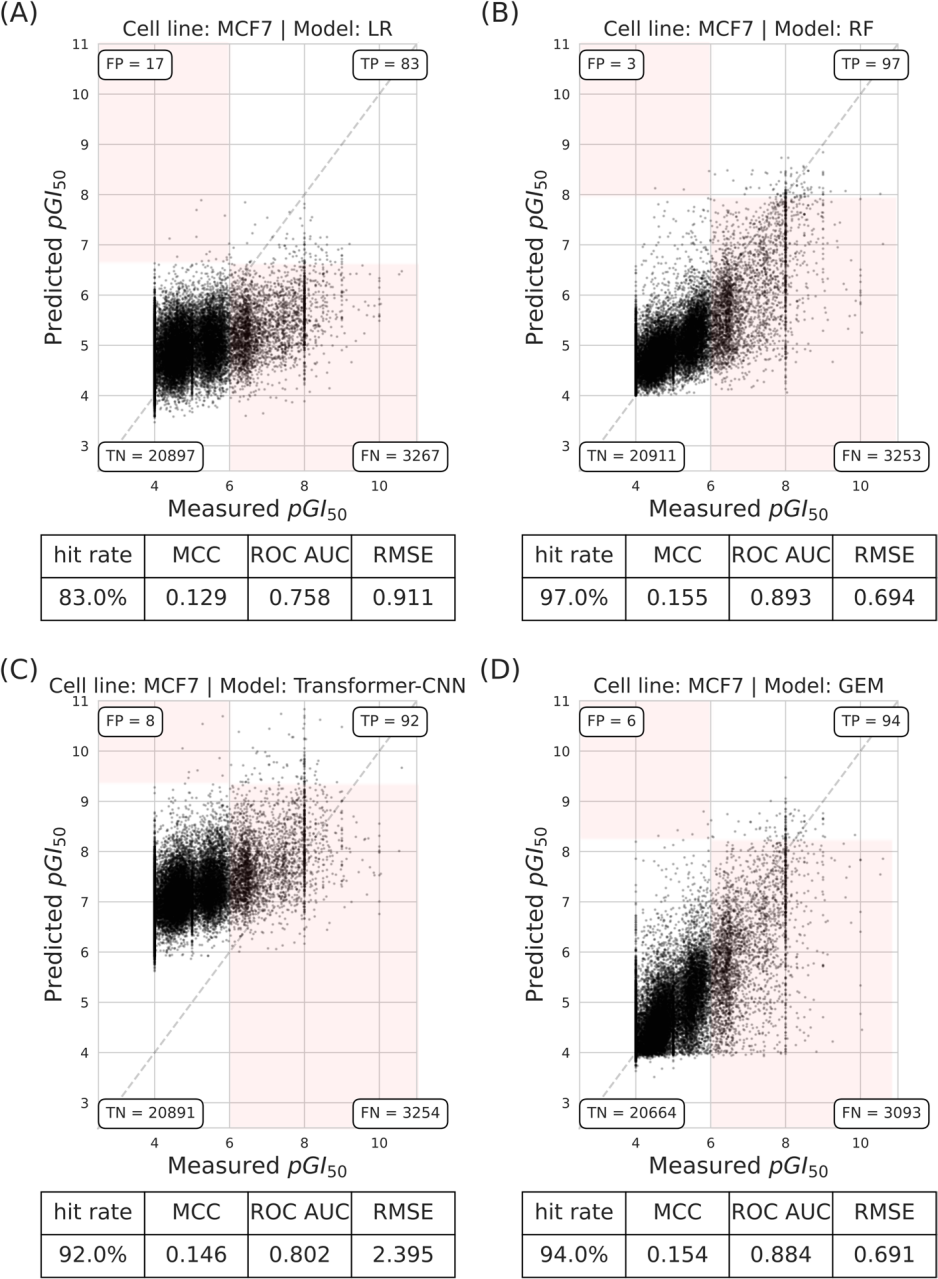
RF and GEM models shows superior hit rates and similar MCC in predicting $pGI_{50}$ values with the random split. The scatter plot showing predicted versus measured $pGI_{50}$ of molecules tested on the MCF7 cell line using the random split with three different regression models. **a** LR. **b** RF. **c** Transformer-CNN. **d** GEM. These results are from the sevenfold cross-validation predictions based on a single seed. Each plot contains the performance metrics for model comparisons

Figure 3 shows the relationship between predicted and measured $pGI_{50}$ values for molecules using random split. The unusual concentration of molecules at measured $pGI_{50} = 4$ is due to the inhibitory activity of some weaker molecules being rounded up at the closest considered $pGI_{50}$ of 4 (this is also the case for some molecules more potent than measured $pGI_{50} = 8$). The RF model achieved the highest hit rate at 97%, closely followed by GEM, with a 94% hit rate. In contrast, the LR model demonstrated a more modest hit rate of 83%. Transformer-CNN achieves a hit rate of 92%, outperforming LR but trailing RF and GEM. For Matthews Correlation Coefficient (MCC), RF and GEM have nearly identical values (0.155 and 0.154, respectively), slightly higher than that of Transformer-CNN (0.146). These low MCC values are due to the large number of FN predictions from VS methods, as they aim at reducing the number of FP predictions.

**Fig. 4:**
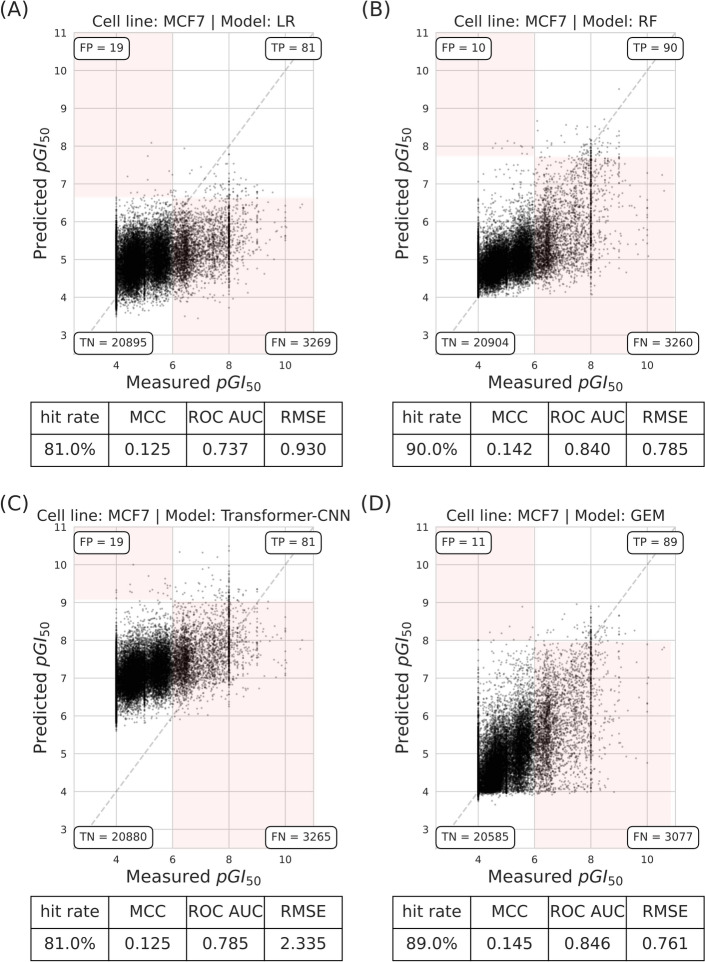
All models show a slight performance drop with scaffold splits with respect to random splits. The scatter plot shows predicted versus measured $pGI_{50}$ of molecules tested on the MCF7 cell line using the scaffold split with different regression models. **a** LR. **b** RF. **c** Transformer-CNN. **d** GEM. These results are from the sevenfold cross-validation predictions based on a single seed. Each plot contains the performance metrics for model comparisons

The scaffold split results (Fig. 4) were similar to those of the random split, with RF and GEM performing better than LR in terms of hit rate. However, the MCC shows an inverse trend, with GEM slightly better (0.145) compared to RF (0.142). Transformer-CNN and LR perform less favorably, with hit rates of 81% and MCC values of 0.125 for both. Overall, the models experienced a slight decrease in hit rates under the scaffold split, indicating that while this method introduces additional challenges, it may not be rigorous enough to fully differentiate their capabilities.

GEM and RF demonstrated comparable performance across both random and scaffold splits, suggesting that while GEM is a more complex model, it does not provide a substantial improvement over the simpler RF model in this context. Despite achieving the highest MCC and slightly better ROC AUC when using scaffold split, GEM’s performance gain does not justify its complexity, particularly when RF achieves nearly the same results with lower computational demands. In this context, GEM’s added complexity does not translate into a significant advantage.

**Fig. 5:**
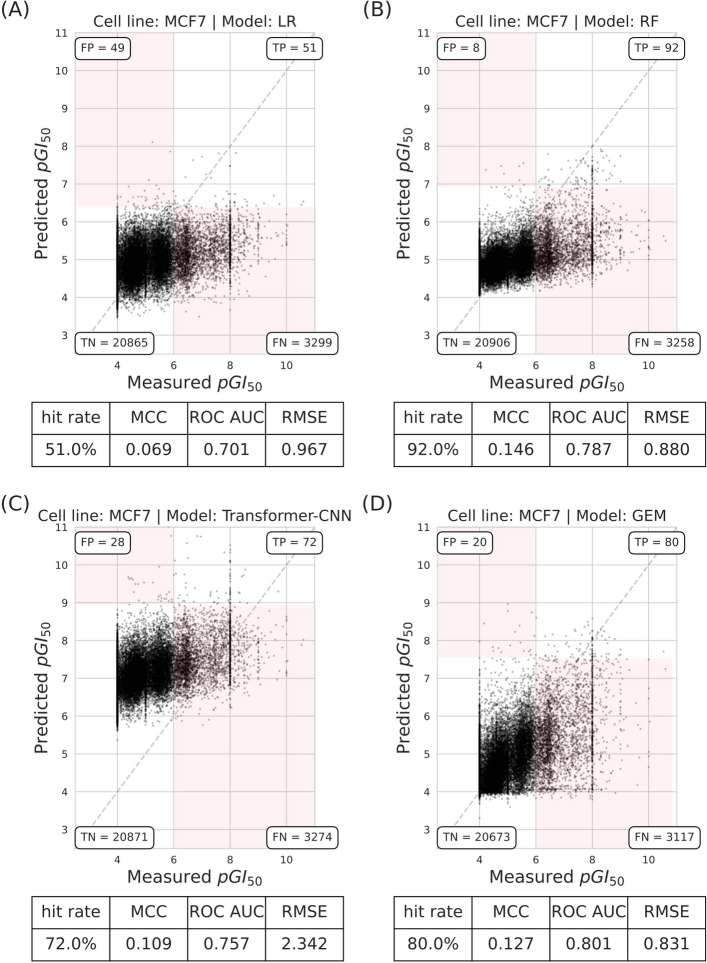
Disagreement between hit rate and ROC AUC with the Butina split highlights the importance of metric selection in VS. Predicted $pGI_{50}$ vs measured $pGI_{50}$ of molecules on the MCF7 cell line using Butina split with three different regression models. **a** LR. **b** RF. **c** Transformer-CNN. **d** GEM. These results are from the sevenfold cross-validation predictions based on a single seed. Each plot contains the performance metrics for model comparisons

Additionally, under Butina split, RF achieved a higher hit rate than GEM, making it the preferable model compared to GEM, Transformer-CNN and LR, as demonstrated in Fig. 5. Although the ROC AUC scores for RF and GEM were similar, the hit rate difference of 12% highlights the divergent focuses of these two metrics and their impact on model evaluation. Transformer-CNN exhibits a moderate hit rate of 72%, while LR lags significantly with a hit rate of 51%. These results also validate why using more suitable metric, such as the hit rate, is important for VS, where early identification of active compounds is key.

### GEM outperforms RF when using the more realistic UMAP clustering split

**Fig. 6:**
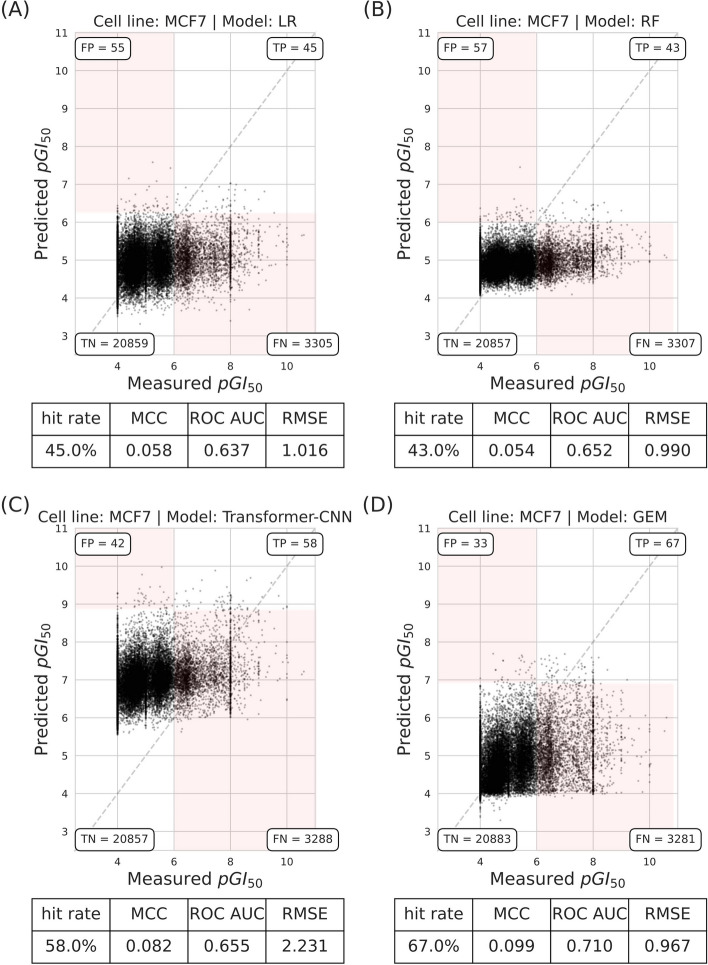
GEM exhibits superior generalizability in VS when using the more realistic UMAP clustering split. Predicted $pGI_{50}$ *versus* measured $pGI_{50}$ of molecules on the MCF7 cell line using UMAP clustering split with three different regression algorithms. **a** LR. **b** RF. **c** Transformer-CNN. **d** GEM. These results are from the sevenfold cross-validation predictions based on a single seed. Each plot contains the performance metrics for model comparisons

Figure 6 illustrated the results with the UMAP clustering split, which presents a significantly more challenging benchmark compared to traditional splitting methods. Both LR and RF exhibit a massive drop in performance, with RF’s hit rate falling from an average of 93–43%. While GEM also performed worse with UMAP clustering split, it achieved the highest hit rate (67%) on the MCF7 dataset.

The results showed that the Butina split results in a substantially easier benchmark than the UMAP split, consistent with the low clustering quality for the NCI-60 dataset [24]. While both clustering methods create a more challenging evaluation, UMAP split is the more effective choice for evaluating VS models, as it better mirrors the complexity and variability of real-world datasets.

Compared to the traditional splits on the same dataset, these results highlight the importance of selecting the appropriate computational tools and evaluation strategies for VS. GEM’s generalizability and predictability under the more realistic UMAP clustering split demonstrated its potential to identify active compounds with novel chemical structures for the MCF7 cell line. Thus, we can better assess the true predictive capabilities of VS models through the UMAP clustering split.

### Robust performance across 60 datasets demonstrates GEM’s superiority

**Fig. 7:**
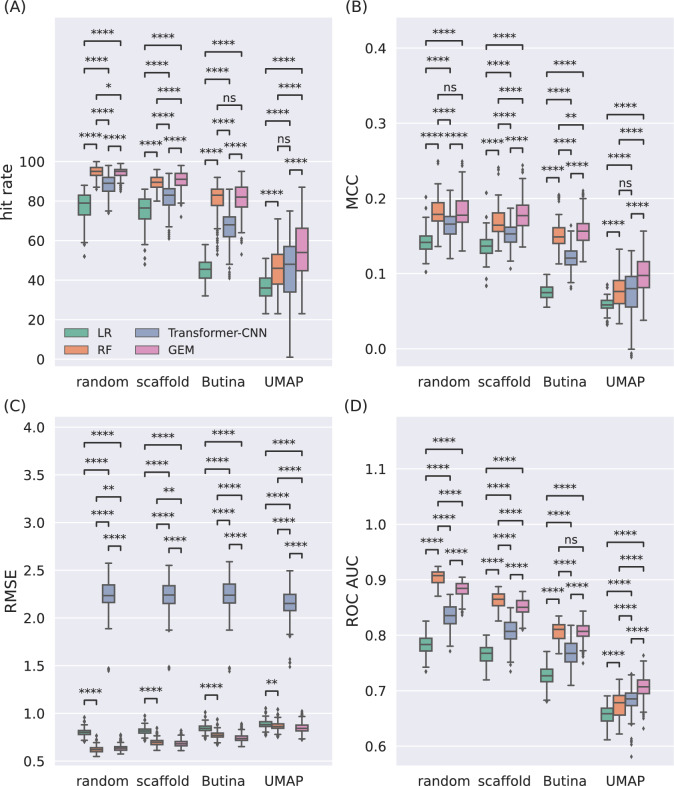
The results that based on merged predictions across 7 cross-validation folds. When the more realistic UMAP clustering split was used, GEM’s median score significantly outperformed RF in all performance metrics. Cross-validation results for the three regression models across the sevenfold cross validation, 60 datasets and 5 seeds with each splitting method. Performance was evaluated with four metrics: **a** Hit rate. **b** MCC. **c** RMSE. **d** ROC AUC. *P*-value $\left( p \right)$ annotation legend: ns $\left( {5.00 \times 10^{ - 2} < p \le 1.00 \times 100} \right)$, * $\left( {1.00 \times 10^{ - 2} < p \le 5.00 \times 10^{ - 2} } \right)$, ** $\left( {1.00 \times 10^{ - 3} < p \le 1.00 \times 10^{ - 2} } \right)$, *** $\left( {1.00 \times 10^{ - 4} < p \le 1.00 \times 10^{ - 3} } \right)$, **** $\left( {p \le 1.00 \times 10^{ - 4} } \right)$

To assess the generalizability of our findings, we extended our evaluation to all 60 datasets (Fig. 7), observing trends consistent with those from the MCF7 cell line (Figs. 3, 4, 5 and 6). The cross-validation results indicate that model performance varies significantly depending on the data splitting method used.

When using the random and Butina splits, RF is the preferable model as it has a higher median hit rate than the other two models. RF consistently outperformed GEM and Transformer-CNN in this metric, despite GEM performing better in terms of median RMSE scores under Butina split. However, in VS applications, the hit rate is deemed a much more pertinent metric for VS due to its emphasis on false positives (these have the highest priority in practical VS applications).

Conversely, the UMAP split assessment, offering a more realistic evaluation, revealed that the median score of GEM strongly outperformed RF and Transformer-CNN in all performance metrics. GEM’s superior median scores indicate its increased efficacy in more realistic VS tasks requiring robust generalization.

Notably, UMAP split results corrected the misleading conclusions drawn from random and Butina splits, underscoring GEM as the optimal model for identifying active compounds under realistic conditions. Results with either of these splits would have led to the incorrect conclusion that RF was the optimal model based on the median hit rate performance. However, UMAP split results show that the GEM model is a more suitable choice. These results corroborate our hypothesis that the choice of splitting method can influence the model evaluation outcomes, and that UMAP clustering split provides a more accurate real-world performance.

Transformer-CNN showed intermediate performance across splits. While it outperformed LR consistently, it trailed both GEM and RF under random, scaffold, and Butina splits. However, under the UMAP split, Transformer-CNN’s generalization improved, with an increase in hit rate compared to simpler splits, though it still lagged behind GEM. These results suggest that Transformer-CNN’s architecture allows it to capture complex relationships in challenging datasets, but additional optimization is required for it to match GEM’s performance.

The solidity and reliability of these model performance results are further strengthened by their statistical significance. Differences between the models across the splitting methods are highly significant, with strong effect size across all cases in Fig. 7. Each of the boxplots in Fig. 7 is formed by sevenfold merged cross-validation using 5 different seeds for all the 60 cell lines ($5 \times 60 = 300$ evaluations) of the considered metric. This assessment ensures that the performance differences observed are robust and not attributable to random variation.

### ROC AUC is unable to select models providing the highest hit rate

Our findings indicate that the widely reported evaluation metric, ROC AUC score, cannot navigate to the model with better hit rate. We consider 300 sets of sevenfold merged cross-validated predictions and their calculated ROC AUCs and hit rates. These sets come from 5 seeds and 60 cell lines using GEM with UMAP splits.

**Fig. 8:**
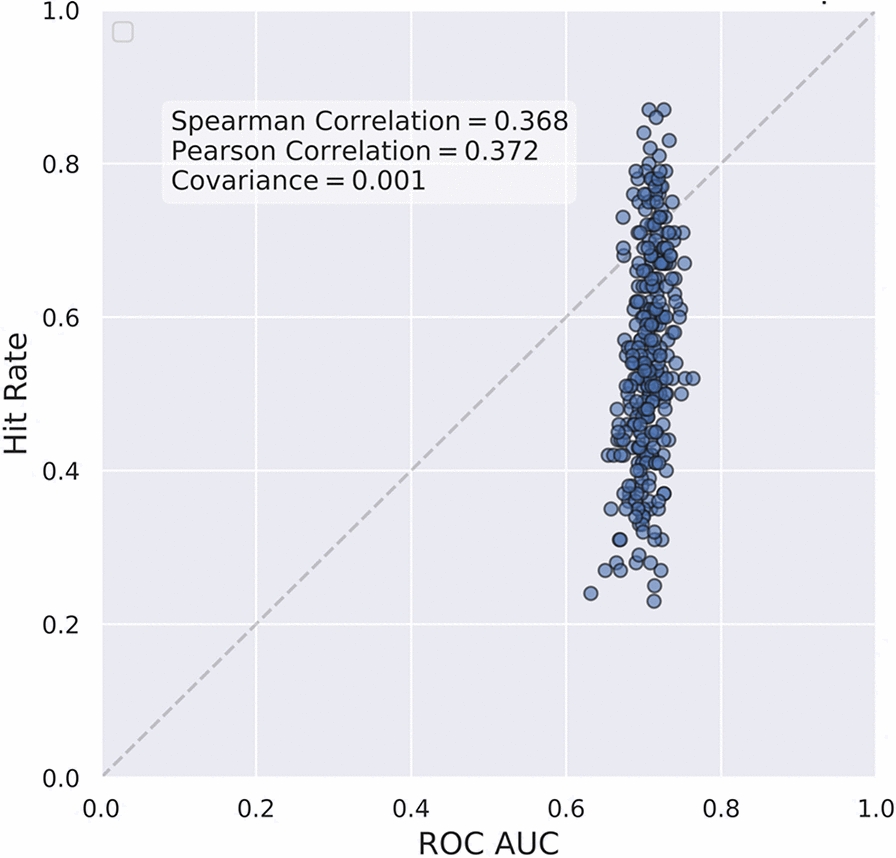
Correlation between ROC AUC and hit rate for GEM on UMAP split. The figure illustrated weak correlation between ROC AUC scores and hit rates across evaluated models. Each point represents one seed model’s performance for one cell line, showing that similar ROC AUC scores can correspond to vastly different hit rates

We calculated Spearman rank correlation and Pearson correlation between both metrics across the 300 sets, observing weak correlations of 0.368 and 0.372, respectively. This is evident from Fig. 8, where models with ROC AUC around 0.7 offer hit rates approximately ranging from 20 to 80%. This result shows that ROC AUC is unable to discriminate between models with high and low hit rates. Thus, many benchmarks using ROC AUC on their leaderboards are identifying models that are unlikely to have any value for early-recognition problems such as VS.

## Discussion and conclusion

Our study demonstrated the importance of employing realistic data splitting methods for evaluating AI models in VS, particularly when predicting drug activities against cancer cell lines. Traditional splitting methods such as random and scaffold splits are widely used but are insufficient for simulating real-world VS scenarios. They tend to overestimate model performance by not adequately accounting for the vast chemical diversity and dissimilarity of compounds encountered in practical applications.

The scaffold and Butina splits are often misconceived as able to mimic performance in real-world scenarios. Although they introduce some dissimilarity among molecules, compounds within the same groups often retain high levels of similarity [57]. This limitation undermines their ability to evaluate a model’s capability to identify truly novel structures. Identifying such a genuinely novel structure is important for discovering new therapeutic pathways and overcoming drug resistance. In contrast, the UMAP clustering split method simulates better this real-world situation, by effectively clustering molecules based on their chemical similarity. This approach ensures that dissimilar molecules are assigned to different sets to evaluate the model’s generalizability, which is more aligned with the goal of patentable drugs with novel scaffolds. This method better reflects the challenges of identifying patentable drugs with novel scaffolds. For example, GEM median hit rate with the Butina split approximately doubles that with the UMAP split, demonstrating the increased difficulty of the latter.

We prioritized hit rate as the primary metric and MCC as the secondary metric, aiming to reduce false positive errors in the early drug discovery stage, which are critical for identifying potential drug leads. The hit rate focuses on reducing FP errors. The secondary metric MCC measures both FN and FP errors. While the most reported performances in machine learning literature are ROC AUC for classification and RMSE for regression tasks, these metrics are less suitable for VS tasks. While ROC AUC has desirable statistical properties, it often fails to prioritize top-ranking actives, as required in practical VS where only the highest-scoring molecules are experimentally tested [58]. This is because they consider errors from all test molecules rather than focusing on those highly ranked ones [58].

When using the Butina split, the GEM outperforms RF in terms of median RMSE scores across the 60 problem instances. In contrast, when using early recognition metrics, specifically hit rate, the Butina split median performance of GEM is slightly worse than RF. Transformer-CNN’s hit rate was lower than RF but higher than LR, reflecting its ability to capture non-linear relationships. When moving on to the UMAP clustering split, GEM’s and Transformer-CNN’s median performances surpassed RF in both ROC AUC and hit rate. However, note that: (a) GEM performance declined under more realistic splits, suggesting an overestimation, though still significantly better than RF, LR, or Transformer-CNN. (b) The performance of RF is indistinguishable from LR under UMAP split, with both median performances being at a random level. (c) Transformer-CNN demonstrates its capacity to handle complex relationships but indicates room for further optimization to achieve parity with GEM under realistic conditions. These findings not only show the limitations of traditional models for prospective VS against cancer cell lines, but also highlight the capabilities of deep learning models in generalizing to dissimilar molecules in this problem. By highlighting the limitations of traditional data splitting methods and demonstrating the enhanced generalization capabilities of GEM under realistic conditions, we emphasize the critical need for advanced modeling approaches in VS. Adopting such models can significantly improve the discovery of active compounds with novel chemical structures, ultimately accelerating the development of new therapeutic agents.

In conclusion, none of the algorithms were performing as well as they were expected. The application of the UMAP clustering split, however, revealed the efficacy of the model in more realistic scenarios, showing the reasons behind the limited success of AI in the drug discovery field and its ability to identify potential drugs.

Furthermore, situating our work within the broader field, specifically for the NCI-60 dataset, reveals a gap in research focused on unseen molecule prediction. Not many studies focus on unseen molecule prediction as our study but instead focus on unseen cell line prediction [22, 59]. In our study, we evaluated the model on unseen molecules, which is fundamentally different from those studies and are not directly comparable to studies across different cell lines [60]. Moreover, there are a few studies in the same domain predicting drug responses using NCI-60 data, these studies primarily relied on random splits [61, 62] or at most scaffold splits [63] or did not proceed to make predictions [64]. Our new approach, employing the UMAP clustering split permits evaluating the generalizability problem in drug discovery not just how the model performs across the biological context (i.e., on different cell lines), but also how the machine learning model can discover novel drug-like chemicals.

Our study has revealed the issue of the commonly used traditional splits overestimating model performance, and UMAP clustering split is a more realistic splitting method. Following proof-of-concept work showing that graph neural networks are particularly suited for VS against cancer cell lines [65]. We have demonstrated here the important limitations of employing the traditional splits on VS problems. As those splitting method will introduce strong training-test similarities regardless of the label to predict, we also expect this split to overestimate model performance in molecular property prediction problems other than VS. This issue will be particularly acute with ultra-large libraries, which can potentially be larger than $10^{20}$ make-on-demand molecules [66]. Traditional splits fail to account for the diversity and novelty inherent in these libraries, as they primarily rely on labeled molecules from in-stock libraries—the standard acquisition method until recently. By not adequately challenging models with truly unseen and diverse chemical structures, traditional evaluation methods may lead to overconfidence in a model’s predictive capabilities. Ultimately, our study emphasizes the urgent need to move currently used evaluation methods to embrace data splits and metrics that reflect the realities of contemporary drug discovery challenges. While the best model on the hardest split achieves very high hit rates, it is important to keep in mind that this type of distribution shifts is only one of the challenges that must be overcome [67].

## Future work

While our study provides valuable insights, several limitations should be considered. First, despite UMAP clustering split offering a more realistic benchmark, it still relies on pre-defined similarity measures, which may not fully capture the complex relationships between chemical structures and bioactivity. Future work could explore alternative clustering methods that incorporate additional molecular properties beyond structural similarity. Second, the NCI-60 dataset, while being the largest to date in this sense, does not encompass the full chemical diversity found in broader drug discovery efforts. Expanding evaluations to larger, more diverse datasets could further validate the robustness of these models. Third, our approach uses ranking-based evaluation metrics, such as hit rate, which better align with VS goals. However, the selection of the top 100 ranked compounds as predicted positives is a reasonable choice but not the only one that could be made. Further research could investigate alternative ranking strategies that dynamically adjust based on dataset characteristics or experimental constraints.

## Data Availability

The code to reproduce the presented results, including preprocessing of the employed public datasets, is available at https://github.com/Rong830/UMAP_split_for_VS.

## Footnotes

1 The URL for downloading this version of NCI-60 data is: https://wiki.nci.nih.gov/display/NCIDTPdata/NCI-60+Data+Download+-+Previous+Releases.

2 The pre-trained parameters can be found at https://baidunlp.bj.bcebos.com/PaddleHelix/pretrained_models/compound/pretrain_models-chemrl_gem.tgz.
